# Author's Reply: Critical Limitations in Systematic Reviews of Large Language Models in Health Care

**DOI:** 10.2196/82729

**Published:** 2025-09-24

**Authors:** Andre Python, HongYi Li, Jun-Fen Fu

**Affiliations:** 1 Center for Data Science, Zhejiang University, Hangzhou, China; 2 School of Medicine, Zhejiang University, Hangzhou, China; 3 Centre for Human Genetics, Nuffield Department of Medicine, University of Oxford, Roosevelt Drive, Oxford, OX3 7BN, United Kingdom, 44 01865 287500; 4 School of Mathematical Sciences, Zhejiang University, Hangzhou, China; 5 School of Medicine, Children’s Hospital of Zhejiang University, Hangzhou, China; 6 National Clinical Research Center for Child Health, Hangzhou, China; 7 National Regional Center for Children's Health, Hangzhou, China

**Keywords:** large language model, LLM, clinical, artificial intelligence, AI, digital health, LLM review, review, letter

##  Introduction

We thank the correspondent for engaging with our original work [1] and raising constructive points in their Letter [2].

## Citation Threshold Bias

We acknowledge that the citation criteria applied to select journals may exclude relevant studies from emerging or specialized venues. Our criteria were not only desirable but necessary to balance comprehensiveness with methodological quality considering the rapidly expanding literature. To mitigate the risk of omission of innovative research, we (1) screened and incorporated all relevant articles from main database platforms as well as e-prints and (2) made available an interactive online guideline offering an up-to-date guide to clinicians.

## Definition of “Best Performance”

We acknowledge the concerns associated with the performance comparison of models across heterogeneous contexts. To avoid ambiguity and misinterpretation, we stated and discussed in detail that, in our study, the term “best performance” is solely associated with the findings from the reviewed studies. Our analysis helps identify models successfully applied in clinical studies, without aiming at or implying comparison across domains. We direct readers to the excellent recent work by Liu et al [3] for a comparison of lightweight large language models (LLMs) for medical tasks.

## Quality Assessment of the Included Studies

We carried out a thorough quality assessment following PRISMA guidelines [4]. This might have escaped the correspondent’s attention, as the details are provided in Multimedia Appendix 2 of our work [1].

## Clinical Workflow

The suggested 5-stage workflow does not ignore nor intend to capture the complexity of clinical practice. Rather, it serves as a framework to associate the reported use of LLMs with tasks and processes familiar to clinicians, in line with a previous study [5]. Our workflow offers a practical assessment of the role and extent of LLMs applied in clinically relevant sectors of activities and tasks.

## Clinical Validation Gap

We acknowledge and discuss the challenges in assessing the practicality of their deployment in clinical applications. Complementary to benchmarking LLMs on research datasets, our review covers studies using LLMs in both research and clinical settings. While we identified key challenges of LLMs in real-world applications, a comprehensive assessment of discrepancies between research and clinical settings is clearly beyond the scope.

## Safety and Risk Analyses

While our review discusses key concerns of the use of LLMs in clinical settings including hallucination risks and ethical considerations, a comprehensive risk assessment is beyond scope. Future research dedicated to tackle this key topic would require substantial efforts.

## Economic Evaluation

Our review assesses the associated costs of the graphics processing unit memory and its cooling requirements by process and clinical tasks. Our interactive online guideline will regularly incorporate future changes in the requirements and costs, as exemplified by the recent rise of lightweight LLMs that may offer excellent performance on consumer-grade hardware. However, a comprehensive cost-effectiveness or return-on-investment analysis is beyond the study scope.

## Conclusion

These observations are a timely reminder that our current understanding of the application of LLMs in clinical settings remains provisional and that we need continual reassessment of their current and future roles in health care practice.

## Abbreviations

LLM: large language model
